# Exploring the Correlation Between the Molecular Structure and Biological Activities of Metal–Phenolic Compound Complexes: Research and Description of the Role of Metal Ions in Improving the Antioxidant Activities of Phenolic Compounds

**DOI:** 10.3390/ijms252111775

**Published:** 2024-11-01

**Authors:** Zhe Chen, Renata Świsłocka, Renata Choińska, Krystian Marszałek, Aleksandra Dąbrowska, Włodzimierz Lewandowski, Hanna Lewandowska

**Affiliations:** 1 Prof. Wacław Dąbrowski Institute of Agricultural and Food Biotechnology—State Research Institute, ul. Rakowiecka 36, 02-532 Warsaw, Poland; zhe.chen@ibprs.pl (Z.C.); r.swislocka@pb.edu.pl (R.Ś.); renata.choinska@ibprs.pl (R.C.); krystian.marszalek@ibprs.pl (K.M.);; 2 Department of Chemistry Biology and Biotechnology, Bialystok University of Technology, Wiejska 45E, 15-351 Bialystok, Poland; w.lewandowski@pb.edu.pl; 3 School of Health & Medical Sciences, University of Economics and Human Sciences in Warsaw, Okopowa 59, 01-043 Warsaw, Poland; 4 Centre for Radiation Research and Technology, Institute of Nuclear Chemistry and Technology 16 Dorodna St., 03-195 Warsaw, Poland

**Keywords:** phenolic compounds, metal–phenolic compound complexes, synthesis, characterization, bioactivities, antioxidant properties

## Abstract

We discussed and summarized the latest data from the global literature on the action of polyphenolic antioxidants and their metal complexes. The review also includes a summary of the outcomes of theoretical computations and our many years of experimental experience. We employed various methods, including spectroscopy (FT-IR, FT-Raman, NMR, UV/Vis), X-ray diffraction, thermal analysis, quantum calculations, and biological assays (DPPH, ABTS, FRAP, cytotoxicity, and genotoxicity tests). According to our research, the number and position of hydroxyl groups in aromatic rings, as well as the delocalization of electron charge and conjugated double bonds, have a major impact on the antioxidant effectiveness of the studied compounds. Another important factor is metal complexation, whereby high ionic potential metals (e.g., Fe(III), Cr(III), Cu(II)) enhance antioxidant properties by stabilizing electron charge, while the low ionic potential metals (e.g., Ag(I), Hg(II), Pb(II)) reduce efficacy by disrupting electron distribution. However, we observed no simple correlation between ionic potential and antioxidant capacity. This paper gives insights that will aid in identifying new, effective antioxidants, which are vital for nutrition and the prevention of neurodegenerative illnesses. Our results outline the connections between biological activity and molecular structure, offering a foundation for the methodical design of antioxidants. Our review also shows in detail how we use various complementary methods to assess the impact of metals on the electronic systems of ligands. This approach moves beyond the traditional “trial and error” method, allowing for the more efficient and rational development of future antioxidants.

## 1. Introduction

Polyphenolic compounds (PPs), widely present in plants, are crucial components of the human diet highly valued for their antioxidant properties [1]. PPs constitute a large class of secondary metabolites found mostly in plants, fungi, and bacteria [2]. With over 8000 identified to date, most known polyphenolic compounds are derived from plant sources like fruits, vegetables, grains, and medicinal herbs [3]. They play essential roles in plant development, defence, and ecological interactions [4]. Phenolic compounds are synthesized mainly through the shikimate pathway, present in plants, fungi, and bacteria and notably absent in mammals. This pathway begins with the condensation of phosphoenolpyruvate and erythrose 4-phosphate, leading to shikimic acid and subsequently producing the aromatic amino acids phenylalanine, tyrosine, and tryptophan. Phenylalanine further serves as the primary precursor for polyphenol production via the phenylpropanoid pathway [5,6]. Beyond the shikimate pathway, polyphenolic compounds can also be synthesized through the polyketide (acetate–malonate) pathway [7]. The polyketide pathway, present in plants, fungi, and bacteria, uses acetyl-CoA or malonyl-CoA to form diverse phenolic structures, including antibiotics, pigments, and toxins. Polyketide-derived polyphenols, such as aflatoxins in fungi, often provide defence mechanisms and support survival under stress, finding applications in pharmaceuticals due to their strong biological activities [8,9]. Fungal polyphenols like orsellinic acid and griseofulvin, synthesized via this pathway, contribute to fungal resilience and facilitate ecological interactions, often demonstrating antimicrobial properties that aid in competitive survival [10,11]. Major groups of polyphenols include, inter alia, flavonoids, phenolic acids, lignans, and stilbenes, each serving distinct functions: flavonoids protect against UV radiation and attract pollinators, phenolic acids strengthen cell walls to resist pathogens, lignans provide structural support, and stilbenes offer antifungal defences. Tannins bind to proteins and reduce the digestibility of plant tissues, making them less appealing to herbivores. Some flavonoids and other phenolic compounds can deter herbivores with their bitter taste, while some polyphenols are directly toxic to insects. In terms of mass, polyphenols can constitute up to 5–10% of the dry weight of many plant tissues, especially in leaves, fruits, seeds, and bark [12,13,14,15].

From the structural point of view, the characteristic feature of a PP is a benzene ring bonded to one or more hydroxyl groups [16]. Depending on the number and overall polarity of their functional groups, they are categorized into water-soluble and water-insoluble types [17]. Due to their potential to prevent and treat certain diseases, phenolic compounds are increasingly appealing as constituents of food products [18]. Many studies suggest their significant health benefits, including antioxidant, anti-ageing, antimicrobial, anti-cancer, and anti-inflammatory effects. They protect various human body systems, including cardiovascular, neurological, renal, and pulmonary systems [19,20]. For example, quercetin has shown a remarkable ability to inhibit bacterial growth and combat viruses, including the HSV-2, respiratory syncytial virus, and poliovirus [21]. Similarly, rutin has proven effective against viruses such as rabies, dengue, and influenza [22]. These properties render phenolic compounds invaluable not only in the food industry but also in pharmaceutical applications.

Phenolic compounds are commonly regarded as natural antioxidants, critical in neutralizing free radicals and reducing the risk of various diseases [23]. The antioxidant capacity of phenolic compounds depends significantly on their molecular structure, such as the number and position of hydroxyl groups on the aromatic ring and the location of double bonds within the C-chain [24]. These structural variations affect antioxidant mechanisms like hydrogen atom transfer, single electron and proton transfer, and sequential proton loss electron transfer, influencing their antioxidant activities [25]. Nevertheless, some phenolic compounds, such as flavonoids, have low bioavailability due to poor solubility, extensive metabolism, and degradation in the gut. Additionally, they are vulnerable to environmental factors such as pH, light, heat, and harsh conditions, which can lead to degradation [26]. To address these challenges, researchers have explored the use of metal ions to coordinate with phenolic compounds, enhancing both their bioavailability and stability. This coordination has been shown to increase phenolic compounds’ antioxidant activities. The coordination of antioxidant ligands with metals with high ionic potential measured by the charge-to-radius ratio (such as Fe(III), Cr(III), Ln(III), Y(III), and Mg(II)) significantly increased the antioxidant properties of these ligands. The decisive factor currently regarded as crucial for this improvement is the delocalization of the ligand’s electronic charge. Metals with low ionic potential (such as Hg(I), Hg(II), and Ag(I)) that disturb the distribution of the electronic charge reduce the antioxidant capacity of the ligands [27]. For instance, the 2,2-diphenyl-1-picrylhydrazyl (DPPH) radical scavenging activity of luteolin after Al(III), Fe(III), and Cu(II) ions’ coordination increased from 1% to 21%, 13%, and 45%, respectively [28]. Extensive studies have revealed that metal ion coordination alters the charge distribution, bond angle, and bond length within phenolic compounds, impacting their capacity for electron and proton transfer, and enhancing their antioxidant properties [27,29,30,31].

Although extensive characterization methods and theoretical computations have been employed to understand changes in the molecular structure of phenolic compounds after metal coordination, the relationship between molecular structure and biological activities has rarely been analyzed. Herein, the review systematically explores the characterization and biological activities of metal–phenolic compound complexes (MPCCs) to understand the connection between structure and activity. We aim to understand how their structural characteristics influence their effectiveness at the molecular level. This correlation is crucial for advancing their application in fields such as medicine, environmental remediation, and food science, potentially leading to the development of more targeted and efficient therapeutic and functional materials.

## 2. Synthesis and Solubility of Metal–Phenolic Compound Complexes

**Figure 1:**
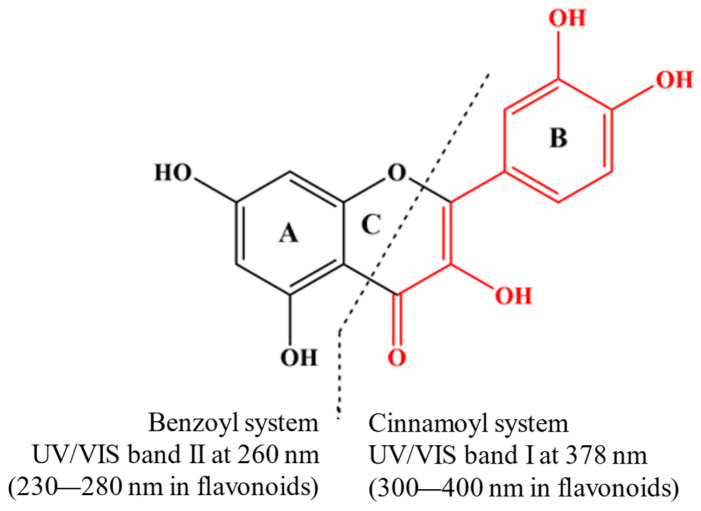
Quercetin is a model compound for investigating molecular changes in metal–flavonoid complexes. Its UV/Vis spectrum shows two leading absorption bands at 378 nm (Band I) and 260 nm (Band II), both attributed to π-π* transitions (Nakamura et al., 2023) [32].

Phenolic compounds coordinate with metal ions primarily through their hydroxyl (–OH) and carboxyl (-COOH) groups. However, other functional groups, such as catechol and galloyl groups, also play significant roles in this coordination (Figure 1). The general preparation of MPCCs involves straightforward chemical synthesis methods. Chromatographic methods separate phenolic compounds from fruits, vegetables, seeds, or leaves, often treated with NaOH for deprotonation, combined with a metal salt solution, and precipitated (Supplementary Table S1).

## 3. Characterization of Metal–Phenolic Compound Complexes

### 3.1. UV/Vis

The UV/Vis spectra of phenolic compounds are commonly associated with electronic transitions occurring between molecular orbitals of the π-type. These orbitals exhibit varying degrees of extension across the molecular backbone, contingent upon the specified subclass [33]. Interaction with metal ions notably influences these transitions, altering the UV absorption patterns of the phenolic compounds. Quercetin is a model subject for studying the molecular changes in metal–flavonoid complexes. In its UV/Vis spectrum, quercetin exhibits two primary absorption bands at 378 nm (Band I) and 260 nm (Band II), corresponding to π-π* transitions [32] (Figure 1). When metal ions are present, an additional band (Band III) can manifest due to ligand–metal charge transfer (LMCT) transitions or d-d transitions, suggesting additional interactions beyond the basic π-π* transitions. The new absorbance peak (Band III) typically appears between the existing Band I and Band II. For example, forming quercetin–metal complexes with ions like Cu(II), Na(I), Ni(II), and Zn(II) results in a notable bathochromic shift at Band I, while no significant differences are observed at Band II. Notably, the chelation with copper (II) ions is likely to involve the A- or C-ring of quercetin [34]. In the Zn(II)–quercetin complex, significant shifts and changes in absorption intensity are observed [34], attributed to extended conjugation within the complex, possibly involving the formation of a new ring with the metal at the 3-hydroxyl and 4-oxo groups [35]. When complexed with Cu, a new weak shoulder peak (Band III) is noted between the two absorbent peaks of quercetin, which may be caused by the electron transfer from quercetin to the metal centre [36]. The different interactions between quercetin and various metals result in varying redshift levels and absorption intensity changes (Table S1). This variance can be linked to the type of metal–quercetin interaction, whether ionic or covalent. The degree of these changes also depends on the ratio of phenolic compounds to metal, which influences the absorbance at the maximum absorption peak [37], suggesting changes in the coordination mode [38]. The absorbance value of the complexes at the absorption peak increases with the increase in the ratio of phenolic compounds to metal [37]. However, no clear correlation has been established between these spectral shifts and the metal’s atomic or ionic radius, ionization energy, or electronegativity.

### 3.2. Vibrational Spectroscopy (FT-IR, FT-Raman)

Vibrational spectroscopy (FT-IR, FT-Raman) is an essential method for assessing aromaticity and electric charge distribution in aromatic rings. In this case, the spectroscopic criterion of the aromaticity of organic compounds is used: in the ranges of about 1610, 1590, and 1450 cm^−1^, there are intense, narrow bands of the aromatic system. Analysis of the number, intensity, and purity of these bands indicates the stabilization or disorder of the aromatic system of ligands during their complexation by various metals. Metals with high ionic potential stabilize the electronic system of ligands, while metals with low ionic potential disturb this system.

The hydroxyl group (–OH) vibrations in polyphenols are typically visible in two central regions: the O–H stretching vibrations generally appear as a broad band in the region of 3200–3600 cm^−1^. This broadening is often due to hydrogen bonding, common in polyphenols because the hydroxyl groups tend to form intra- or intermolecular hydrogen bonds. In-plane bending vibrations of the hydroxyl group can appear around 1200–1400 cm^−1^, while out-of-plane bending vibrations typically occur in the lower region, around 650–900 cm^−1^. The C–OH vibrations range around 1200–1400 cm^−1^, often coupled with in-plane O–H bending [39]. Additionally, phenolic acids contain a carboxyl group, showing a characteristic absorption at 1750 cm^−1^ for the C=O bond [40] and peaks at 1650 cm^−1^ and 1420 cm^−1^ for symmetric and asymmetric stretching vibration [41]. A notable difference in the FTIR spectrogram between free phenolic compounds and their complexes was observed upon metal ion complexation. The impact of different metals on the FTIR spectra of phenolic compounds is displayed in Supplementary Table S2.

Specifically, metal ions typically coordinate with the carboxylic acid and hydroxyl groups in phenolic acids. This interaction leads to the disappearance of the stretching vibrations of C=O and –OH from the carboxylic acid group while asymmetric and symmetric vibrations of the carboxylate anion appear [42]. These changes suggest that metals coordinate with the carboxylic acid group. It can be seen that, in addition to the carboxyl group, the catechol groups in phenolic compounds also coordinated metal ions. The stretching vibration of the catechol group was noted at 1286 cm^−1^ for chlorogenic acid. In contrast, the bands for chlorogenic acid–Cu and –Fe complexes were shifted to lower wavenumbers (from 1286 cm^−1^ to 1261 cm^−1^ and from 1286 cm^−1^ to 1259 cm^−1^, respectively). The finding showed that the catechol group may coordinate Cu(II) and Fe(III) with chlorogenic acid [43]. Concerning flavonoids, the stretching vibration of C–OH in kaempferol– and quercetin–Pb complexes was shifted from 1379 cm^−1^ to 1351 cm^−1^ and 1350 cm^−1^, respectively [44]. The shift to a lower wavenumber suggested the movement of electrons from the centre of the band to oxygen [44]. Furthermore, the peak of the C=O group in the quercetin–Zn complexes was moved from 1652 cm^−1^ to 1636 cm^−1^, indicating that this group was engaged in coordinating the Zn ion [45]. The enhancement in the wavenumber position in the C-O-C stretching vibration of quercetin–Cu complexes from 1262 cm^−1^ to 1272 cm^−1^ was attributed to higher bond orders caused by Cu coordination [36].

The differences (Δν(COO-) = ν_as_(COO-) − ν_s_(COO-)) between the asymmetric vibration ν_as_(COO-) and symmetric vibration ν_s_(COO-) of the carboxyl group for phenolic compound–metal complexes are utilized to calculate the coordination type of complexes [42,46]. The Δν(COO-) value of Na–phenolic compound complexes (Δν (COO-) Na) is a standard value [46]. When the Δν(COO-) value of MPCCs is higher than the Δν(COO-) Na value, the coordination type of the complexes is monodentate [46]. When the Δν(COO-) value of MPCCs is lower than the Δν(COO-) Na value, the coordination type of the complexes is bidentate [46]. For instance, when caffeic acid was complexed with Eu(III), the Δν(COO-) value of caffeic acid–Eu(III) complexes was 92 cm^−1^ [46]. The value was lower than caffeic acid–Na complexes, demonstrating a bidentate coordination type. Furthermore, after the complexation of p-coumaric acid with Dy(III), Eu(III), and Gd(III), these complexes showed a lower Δν(COO-) value than the Na–p-coumaric acid complex [42]. The results suggested that these complexes’ coordination type was chelated by the carboxyl group.

Transition metal chelation often leads to pronounced changes suggesting strong coordination, as d-orbitals can participate in bonding, which allows them to form complex coordination geometries, engage in covalent bonding with organic ligands, and engage in π-backbonding, factors which profoundly affect the electronic states and hence the infrared (IR) spectra. For example, transition metals (copper (II), zinc (II), nickel (II), and cobalt (II)) affected the IR spectra of cichoric acid by coordinating with the carboxyl groups of the tartaric acid moiety. In the IR spectra of cichoric acid, bands at 1746 cm^−1^ and 1716 cm^−1^, attributed to the carboxyl group, disappeared upon metal binding. This indicates that the metals bond to cichoric acid through the carboxylate group. New bands appeared in the spectra of metal complexes, indicating the formation of carboxylate anions (ν_s_COO^−^ around 1385–1384 cm^−1^, ν_as_COO^−^ around 1630–1605 cm^−1^, β_s_COO^−^ around 868–851 cm^−1^, β_as_COO^−1^ around 521–520 cm^−1^). Bands associated with the aromatic ring (from the caffeic acid moiety) changed in intensity and location due to metal ions’ influence on the ligand’s electronic charge density. Bands related to hydroxyl group vibrations (βOH) in the IR spectrum of cichoric acid were shifted to lower wavenumbers in the spectra of metal complexes, suggesting an interaction between metal ions and hydroxyl groups [47]. Alkali and alkaline earth metals’ interactions with organic ligands were, in turn, predominantly ionic. The metal cations formed electrostatic interactions with negatively charged parts of the molecule (like deprotonated carboxyl groups forming carboxylates). The coordination involved less sharing of electrons, leading to weaker interactions and less dramatic changes in the electronic environments around the ligand’s functional groups. When alkaline metals coordinated and destabilized the aromatic system, the IR spectra showed a decrease in both the aromatic bands’ intensity and the wavenumber values. Lanthanides, like 3d transition metals, typically stabilized the electronic system of the aromatic ring in ligands such as benzoates, salicylates, and 3-pyridine carboxylates by coordinating with the carboxylic group [48]. Lanthanides typically have larger ionic radii and can accommodate higher coordination numbers, often forming complexes that include eight or nine ligands surrounding the metal ion.

### 3.3. 1H and 13C NMR

Nuclear magnetic resonance (NMR) is another essential detection technique used to evaluate the structural information of phenolic compounds after metal ions’ coordination, especially the binding position, thereby verifying the data provided by other detection tools [49]. Compared to free cinnamic acid derivatives, the signal of all protons in complexes was reduced in ^1^H NMR, demonstrating that metal coordination caused a reduction in charge intensity in the aromatic ring, thus elevating its destabilization [50,51]. It was found that the signals on the aromatic ring gradually declined with an increasing radius of metal ions (Li, Na, K, Rb, and Cs) [51]. The findings showed that the double bond between the aromatic ring and the carboxyl group was affected mainly by metal ions. Compared to ferulic acid, homovanillic acid does not include a double bond between the aromatic ring and the carboxyl group. The results showed that the chemical shift of H7 and H8 on carbon between the aromatic ring and carboxyl group did not change in ^1^H NMR. The signals of C7 and C1 between the aromatic ring and carboxyl group declined. The above phenomena demonstrated that when metal ions coordinate with the carboxyl group from cinnamic acid derivatives, they significantly affect the charge density of the atom near the carboxyl group.

Concerning flavonoids, the proton belonging to the OH group from C5 disappeared in Ga(III)–chrysin complexes, suggesting that the hydroxyl group at the C5 position was involved in the coordination of Ga(III) [52]. Meanwhile, the signal of H3 shifted by about 0.2–0.3 ppm, indicating that the coordination of Ga(III) influenced the charge density of H3 at the C3 position [52]. In ^13^C NMR spectra, the signal of C5 in complexes shifted downfield by 4.9 ppm, showing that the carbon atom at position 5 was deshielded, thereby confirming that the C5–OH group was engaged in chelating Ga(III) ions [52]. Compared to chrysin, the disappearance of the proton from the OH group was observed at the C3′ and C4′ positions, which indicated the participation of a catechol moiety in coordinating Pb(II) [44].

### 3.4. X-ray Analysis

The differences in the XRD patterns suggest varying coordination geometries and interactions depending on the metal. For instance, the formation of metal–oxygen bonds is affected by the size and electronegativity of the metal ions, which directly impacts the crystalline properties. Ca(II)–gentisic acid and Mg(II)–isoferulic acid complexes are crystallized in the monoclinic space group C2/c. The asymmetric unit of Ca–gentisic acid complexes has half of lattice water molecules [53], whereas the asymmetric unit of Mg–isoferulic acid complexes has half of three lattice water molecules [54]. The length of the Ca-O bond in the complexes ranges between 2.419 and 2.453 Å [53]. Nevertheless, the length of the Mg-O bond in the complexes ranges from 2.042 to 2.098 Å [54]. The differences are attributed to metal atoms’ different radii and electronegativity (Ca(II) and Mg(II)). Strong hydrogen bonds formed by the carboxylate group and hydroxyl substituents of the aromatic ring interact with coordinated and lattice water molecules to hold neighbouring units together, thereby stabilizing the conformation of the complexes [53,54].

### 3.5. Thermal Analysis

Phenolic compounds as food products are widely applied in the food industries, where pasteurization processing is an important treatment process that enables these products to be safely available on the market. Therefore, the heat stability of phenolic compounds is regarded as a vital attribute to be investigated. Thermogravimetric (TG) and differential scanning calorimetry (DSC) are extensively used as characterization techniques to study the thermal stability of phenolic compounds because of their simplicity and accuracy. From the TG and DSC curves, it can be seen at what time and temperature phenolic compounds appeared to be dehydrated, decomposed, and oxidized by calculating the mass loss of phenolic compounds. Generally, the thermal stability of phenolic compounds after metal coordination was improved. It was found that the decomposition of *p*-coumaric acid occurred at 180 °C and ended at 550 °C [42]. The chelation of p-coumaric acid with Eu, Gd, and Dy metal ions extended its decomposition range up to 900 °C, with maximum decomposition peaks (Tmax) increased by approximately 220 °C, 240 °C, and 240 °C, respectively. Furthermore, the decomposition of caffeic acid was in the range of 200–570 °C, while the decomposition of Eu– and Gd–caffeic acid was in the range of 240–900 °C and 250–900 °C, respectively [42]. The incorporation of lanthanides into cinnamic acid derivatives gave them a more stable form than the free cinnamic acid derivatives. The results mentioned above were attributed to an increase in the intramolecular and intermolecular cross-linking of phenolic compounds caused by the addition of metal ions.

### 3.6. Electrochemical Methods

Cyclic voltammetry (CV) is likely the most widespread voltammetric method for studying redox systems and characterizing antioxidant activity. In a basic CV experiment, the voltage applied on the surface of a working electrode is scanned at a constant rate, while the Faradaic current produced during the oxidation of an antioxidant is recorded. Generally, the voltammogram peak potential gives information on the ease of a molecule to exchange electrons, where the peaks at low oxidation potentials are associated with compounds with a great ability to donate electrons and vice versa [55].

Porfirio evaluated the influence of the complexing on the behaviour of the oxidation peaks characteristic of polyphenol active sites through cyclic voltammetry. The complexation of an Fe (II) ion with the five flavonoids (morin, quercetin, fisetin, catechin, and chrysin) was evaluated from the comparison of cyclic voltammograms obtained in free flavonoids. The information associated with the voltammetric profiles presented by the authors shows that most of the five antioxidant compounds produced the three characteristic oxidation peaks for flavonoid molecules (first peak catechol/pyrogallol (OH(C3, C4)/OH(C3, C4, C5)) on the B ring (the most reactive redox group); second peak hydroxyl (OH) at position C3 on the C ring (difficult to separate from the first peak); and third peak resorcinol (OH(C5, C7)) on the A ring). The oxidation peaks for specific active sites were identified according to prior works [56,57,58]. Chrysin, as it lacked the catechol and hydroxyl groups at position C3, did not produce peaks from their oxidation. The voltammograms for Fe(II)–flavonoid complex formation suggest that morin coordinates with Fe(II) at the site corresponding to the C3 hydroxyl and C4 carbonyl groups. Similarly, quercetin and fisetin present two possible coordinate sites with Fe(II). The first site corresponds to the C3 hydroxyl and C4 carbonyl groups, and the second site corresponds to the catechol group. Due to the presence of at least two of the three active sites responsible for interaction with metal ions, the flavonoids morin, quercetin, and fisetin showed a significant increase in their antioxidant capacity after iron complexation (approximately 15%, 32%, and 28%, respectively). The formation of a stable Fe(II)–phenolic complex involving both the C3/C4 site and the catechol group in metal coordination is evidenced by the presence of a new peak near 300 mV. However, due to the presence of only one complexing active site, which was kinetically unfavourable, the catechin and chrysin Fe complexes did not show any significant differences in their antioxidant capacity [59].

### 3.7. Other Characterization Methods

Besides the characterization approaches above, the structural properties of MPCCs were also studied through elemental analysis, scanning electron microscopy (SEM), and transmission electron microscopy images (TEM). The SEM morphology of gallic acid displayed that particles from gallic acid, after chelating with Cr(III), Ca(II), Cu(II), and Zn(III), were agglomerated to form the different shapes of the bigger particles [60]. The particle shapes of Cr–, Ca–, Cu–, and Zn–gallic acid were small square pieces, small irregular fragments, flat rectangular discs, and small rectangular projections, respectively [60]. Furthermore, the Cr–, Ca–, Cu–, and Zn–gallic acid complexes demonstrated an ordered matrix in the TEM morphology, verifying that they possessed a homogeneous phase material [60].

## 4. Theoretical Computations

### 4.1. Bond Length and Angle

The bond length and angle of MPCCs are essential indices for determining the structural changes in the phenolic compounds after metal coordination. Some studies reported that when phenolic compounds were chelated with metal ions, the bond length of the metal connecting with the O1 from the carboxyl group of phenolic compounds increased [50,51]. Meanwhile, the bond length of C7–C8 in p-coumaric acid, ο-coumaric acid, and homovanillic acid complexes increased, with the order of the increase being Li < Na < K [50,51]. The results showed that when phenolic compounds were coordinated with metal ions, the C-C bond near the metal–O bond in their complexes was affected, and the level of impact increased with the increasing radius of metal ions. The bond length of the aromatic ring in the complexes did not change compared to free ligand acid. When the C=C bond near the metal–O bond appeared in the metal ion–pp-coumaric acid and –ο-coumaric acid complexes, the bond length of the C=C bond in the complexes was lower than that of free pp-coumaric acid and ο-coumaric acid.

Concerning the bond angle, C–O–metal in MPCCs had a lower value than C-O-H in free phenolic compounds [47,50,51]. The bond angle values in C–O–metal increased as the metal ions’ radius (Li, Na, and K) increased [50,51]. Additionally, the bond angle of the O-C-C bond in the carboxyl group of complexes was also increased [50,51], and this angle increased with the radius of metal ions [50,51]. Therefore, the length and angle of the bonds near metal–O were significantly affected after chelating with metal ions.

### 4.2. Dipole Moment, Energy, and Aromaticity

Dipole moment, energy, and aromaticity are crucial factors for understanding the electronic transition in phenolic compounds after metal complexation [61], thereby enhancing the molecule’s polarity. The dipole moment of cichoric acid, after the coordination of Cu(II), Zn(II), Ni(II), and Co(II) ions, was found to increase compared to free cichoric acid [47]. Furthermore, the dipole moment of these complexes increased with the increasing radius of metal ions [50].

Furthermore, the molecular energy levels of MPCCs were lower than those of free ligands [47,50,51]. The energy reduction was attributed to enhanced symmetry in the carboxylate ion. The discrepancy between metal–ο-coumaric acid and metal–mandelic acid complexes may be attributed to differences in their molecular structure. Unlike mandelic acid, ο-coumaric acid possessed a double bond between the alpha and beta carbons in the sidechain, between the aromatic ring and the carboxylic acid group, which diminished the influence of metal ions on the aromatic ring.

### 4.3. Electric Charge Distribution

To further investigate the changes in the amount and position of charge in phenolic compounds after metal ion coordination, the electron charge distribution of the complexes was analyzed using Mulliken, natural bond orbital (NBO), and atomic polar tensor (APT) approaches [62]. Some studies demonstrated that the charges on O and C atoms from the carboxylic acid groups in MPCCs were higher than those in free ligands [47,50,51,63]. The charges of O and C atoms in the complexes increased with the radius of metal ions (Li(I), Na(I), and K(I)) [50,63]. The total charge of the carboxylic acid group in the metal complexes was also found to have increased [50,63]. The charge of the C atom near the carboxylic group in the metal complexes was enhanced compared to free ligands, with values increasing as the radius of metal ions increased [50,51]. Moreover, Samsonowicz et al. [41] indicated that the total charge of the aromatic ring in the Na–homovanillic acid complexes was lower than that of free homovanillic acid. Nevertheless, an increase in the total charge of the aromatic ring was observed in the metal (Li(I), Na(I), and K(I))–ο-coumaric acid and mandelic acid complexes [51,63]. These results demonstrate that the coordination of metal ions significantly impacts the charge distribution in phenolic compounds. Also, the variations in the total charge and the charge on the C atoms of the aromatic ring after metal ions’ chelation are influenced by the positions of substituents on the aromatic ring.

### 4.4. Nephelauxetic Effect

The nephelauxetic effect involves an expansion of the cloud of probability of finding electrons in the central ion. The nephelauxetic effect characteristic of ions with partially filled subshells is caused by the transfer of electrons from ligands to unfilled subshells of the central ion. Donor electrons cause a reduction in the effective charge of the nucleus Z*, reducing its central field. The electrons of the central ion subjected to the reduced, as compared to the isolated ion, effective charge of the nucleus increase the radial dimensions of their orbitals, which weakens the inter-electron interaction. Regarding the above, the greater the positive charge of the central ion and the smaller the radius of atomic orbitals, the greater the nephelauxetic effect. It can be concluded that increasing the ionic potential of the central ion will lower the energy of the complex compound, increasing the antioxidant potential. Moreover, unfilled d and f orbitals will promote stabilization and antioxidant properties. These theoretical considerations correlate with the experimental results discussed here.

Metals with high ionic potential (such as Fe(III), Cr(III), Mg(II), Cu(II), and Zn(II)) stabilize the distribution of the electronic charge, increasing the antioxidant properties of ligands [43,64], and complexation with copper ions leads to significant shifts in absorption, which supports the idea of d-orbital involvement from metals like Cu(II) [34,35,36]. Metals with high ionic potential stabilize the aromatic system of ligands, reducing the distortion of electron charge distribution [43,44,45].

### 4.5. Energy of HOMO and LUMOs

The highest occupied molecular orbital (HOMO) is regarded as an electron donor, while the lowest unoccupied molecular orbital (LUMO) is considered an electron acceptor. HOMO and LUMO indices are commonly used to assess the biological activity and chemical reactivity of phenolic compounds and their complexes. The difference between the HOMO and LUMO (ΔHOMO/LUMO) indicates the reactivity and bioactivity attributes of phenolic compounds. A low ΔHOMO/LUMO is associated with a high biological activity and reactivity of molecules. Samsonowicz et al. demonstrated that the ΔHOMO/LUMO value of homovanillic acid decreased after coordination with Na, suggesting that Na–homovanillic acid complexes exhibit higher biological and reactive activities than the free ligands [50]. Moreover, the ΔHOMO/LUMO values of metal ((Li(I), Na(I), and K(I))–mandelic acid were lower than that of free mandelic acid, and their value gradually declined with the increased radius of metal ions. The reduction in complexes’ ΔHOMO/LUMO values indicates that the salts are more polar and active [63].

### 4.6. Proposed Coordination Mechanism of Complexes

**Figure 2:**
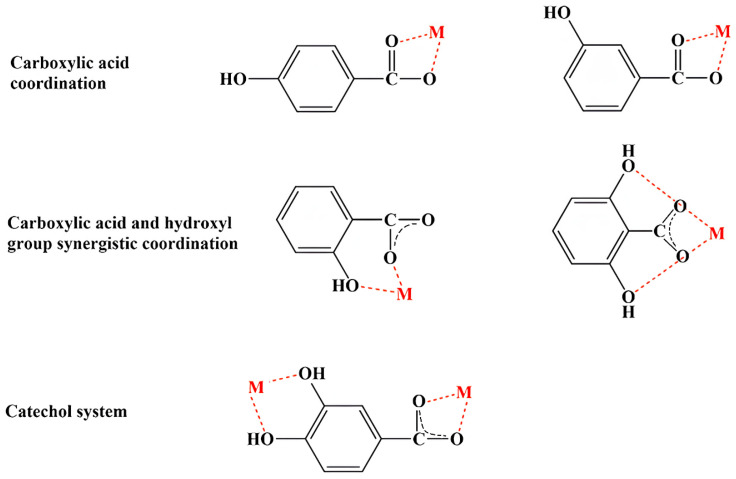
Potential coordination modes of phenolic compounds with metal (M) ions.

Based on the results from various characterization techniques (UV/Vis, FT-IR, 1H and 13C NMR, X-ray analysis, thermal analysis, SEM, and TEM) and theoretical computations (bond length and angle, dipole moment, energy, aromaticity, electron charge distribution, energy of HOMOs and LUMOs), coordination modes of MPCCs have been proposed (Figure 2). These coordination modes primarily involve the carboxyl group and often a synergistic interaction between the carboxyl and hydroxyl groups and the catechol group in phenolic compounds. In the case of flavonoids, which lack a carboxyl group, coordination with metal ions primarily occurs through the synergistic effects of carbonyl and adjacent hydroxyl groups. For example, the mode of chelation of quercetin with Al(III), Fe(III), and Cu(II) involves three different coordination sites, primarily in hydroxyl and carbonyl groups. Al(III) and Fe(III) ions can bind (bidentately) to 4-carbonyl-5-hydroxyl, 3-hydroxyl, and 4-carbonyl or 3′,4′-dihydroxyl groups, while Cu(II) ions avoid the 4-carbonyl-5-hydroxyl site [65].

## 5. Biological Activity

### 5.1. Antioxidant Activity

Phenolic compounds are extensively recognized as promising antioxidants due to their influential free radical scavenging abilities and enhancement of enzyme activity related to antioxidant reactions [66,67]. Furthermore, some studies have reported that MPCCs exhibit superior antioxidant capacities compared to free phenolic compounds (shown in Supplementary Table S3). This improvement is attributed to structural changes in phenolic compounds after metal coordination. For instance, the half maximal inhibitory concentration (IC50) values of 2,2′-azino-bis(3-ethylbenzothiazoline-6-sulfonic acid) (ABTS) and DPPH radical scavenging activities in Zn–gallic acid complexes decreased by 72% and 82%, respectively [68]. On the contrary, some studies reported a reduction in the antioxidant activity of phenolic compounds after the chelation of metal ions. The DPPH radical scavenging activity and ferric reducing antioxidant power (FRAP) of Li– and Na–rutin complexes, recorded as 61%, 57%, 52 μmol/L, and 50 μmol/L, were slightly higher by 3%, 7%, 4 μmol/L, and 6 μmol/L, respectively, than those of free rutin [69].

The enhancement or reduction in antioxidant activity in the phenolic compounds after metal ion chelation is influenced by specific chemical groups, their electric charge distribution, and the ΔHOMO/LUMO value. The arrangement and quantity of chemical groups on the molecular backbone, such as the catechol structure, hydroxyl (–OH) and carboxyl (-COOH) groups, the presence of conjugated double bonds, and the type of aliphatic chains attached to the benzene ring, significantly affect these activities [70]. Gryko et al. [71] reported that the antioxidant activities (DPPH and OH radical scavenging, FRAP, and cupric reducing antioxidant capacity (CUPRAC)) of cinnamic acid derivatives (including cinnamic acid, p-coumaric acid, caffeic acid, and 3,4,5-trihydroxycinnamic acid) improved with an increase in hydroxyl groups on the aromatic ring. However, the ABTS radical scavenging activity and CUPRAC of caffeic acid decreased when combined with an Eu(III) ion compared to free caffeic acid [46]. This decrease was attributed to the coordination of two hydroxyl groups (catechol group) with Eu(III) ions, which reduced the number of available hydroxyl groups, further highlighting their crucial role in antioxidant activity. Nonetheless, phenolic compounds also display pro-oxidant activities [72]. The same chemical groups that confer antioxidant properties can induce pro-oxidant effects in others, even under the same conditions. For instance, a study on five phenols at a concentration of 10 µmol/L demonstrated a positive correlation between the inhibition of deoxyribose oxidation in the Fe^2+^-EDTA-H_2_O_2_ system and the number of –OH groups. Furthermore, a similar correlation was observed in eight other phenols exhibiting pro-oxidant behaviour [70]. When these phenols were coordinated with metal ions, their –OH groups chelated the metal ions, reducing their pro-oxidant activities and enhancing their antioxidant capacities.

The concentration of phenolic compounds influences their redox properties and their antioxidant and pro-oxidant activities [22]. Changes in concentration can alter the antioxidant or pro-oxidant nature and affect the behaviour of specific chemical groups within phenolic compounds [73]. For instance, an increase in the concentration of a polyphenol may decrease the dissociation of its -COOH group on a phenolic ring or hydrocarbon substituent. On the other hand, high concentrations of phenolic compounds promote interaction between individual polyphenol molecules through hydrogen bonding. This interaction can change their chemical availability, reactivity, and accessibility to free radicals [74].

Flavonoid pro-oxidant properties appear to be concentration-dependent [75]. Quercetin and morin increased H_2_O_2_ concentration in human lymphocytes, while naringenin and hesperetin had no detectable effect in the same concentration range [76]. At higher concentrations (100 μM), quercetin, gossypol, and myricetin enhanced hydroxyl radical formation and lipid peroxidation in rat liver microsomes [77]. Quercetin showed protective and pro-oxidant effects in human leukocytes, reducing oxidative DNA damage at lower concentrations (1–50 μM) but increasing damage at 100 μM [78].

The antioxidant mechanism of phenolic compounds primarily operates through the combination of hydrogen atoms and/or single electron transfer from the dissociated hydroxyl groups on the aromatic ring to free radicals, effectively neutralizing them [24,79]. Some studies have demonstrated that the electric charge distribution on the aromatic rings of phenolic compounds shifts significantly after metal ion coordination, increasing with the radius of metal ions [51]. The introduction of metal ions shifts the electronic cloud around the aromatic ring towards the metal ion, reducing the energy required for dissociating hydroxyl groups, thereby boosting the antioxidant capacity of the compounds [27,63]. Another factor influencing antioxidant activity is the difference in HOMO/LUMO values [27,63]. When phenolic compounds form complexes with metal ions, the ΔHOMO/LUMO values decrease, thus enhancing their antioxidant effectiveness [80].

### 5.2. Antimicrobial Activity

Human diseases are predominantly caused by microbial infection [81]. Metal ions like Cu(II), Ru(III), Ga(III), Bi(III), and Ag(I) are commonly employed as antimicrobial agents to suppress microbial growth [82,83,84]. Additionally, phenolic compounds are known for their antimicrobial properties [18]. When these compounds are chelated with metal ions, the complexes demonstrate enhanced antimicrobial activity (shown in Supplementary Table S3). For instance, caffeic acid has been shown to inhibit the growth of *Escherichia coli*, *Bacillus subtilis*, and *Candida albicans* [46]. Complexes of Eu(III) with caffeic acid exhibited greater inhibitory activity effects against *Escherichia coli*, *Bacillus subtilis*, and *Candida albicans* than caffeic acid [46]. Results suggested that the complexes’ minimum inhibitory concentration (MIC) against the above-mentioned bacterial strains was reduced by 69%, 78%, and 83%, respectively [46].

Phenolic compounds exhibit antibacterial activity partly due to their lipophilic nature. This lipophilicity enhances their ability to interact with and penetrate cell membranes, leading to increased membrane permeability. Once inside the cell, phenolic compounds can interact with enzymes, masking their active sites and inhibiting enzymatic reactions. They may also disrupt membrane integrity, leading to the leakage of intracellular components and the inhibition of DNA synthesis [85,86,87,88]. Additionally, phenolic compounds chelate extracellular metal ions, such as K(I) and Na(I), altering cell membrane potentials [85]. They also bind to substances on the cell membrane, impeding transmembrane energy production and transfer, which increases membrane permeability or causes rupture [89]. The resultant increase in the membrane permeability allows phenolic compounds to penetrate cells, where they interact with enzymes, masking active sites, inhibiting enzymatic reactions [89], and impeding DNA synthesis [90]. The antimicrobial activity of these complexes is enhanced when chelated with metal ions because of charge interactions, resulting in significant changes to membrane potential. Arciszewska et al. [46] demonstrated that Eu–caffeic acid complexes more strongly influenced the surface charge of *Escherichia coli*, *Bacillus subtilis*, and *Candida albicans* than free caffeic acid. Moreover, polyvalent cations in the MPCCs had a more substantial chelation capacity with the carboxylic acid groups on the cell membrane, obstructing the transfer of K(I) and Na(I) ions [91]. On the other hand, when the MPCCs enter into the intracellular milieu, metal ions also interact with enzymes and hinder DNA synthesis, significantly increasing bacterial inactivation due to their synergy effect [92]. Furthermore, high concentrations of metal ions from MPCCs within cells can cause protein aggregation, adversely affecting microbial metabolism [93].

### 5.3. Cytotoxic Activity

The cytotoxic activity of metal–phenol complexes is crucial for designing potential pharmaceutical agents [94]. Phenolic compounds, being secondary metabolites, are generally non-toxic, nutritious, and safe [16]. However, metal ions can be harmful and potentially toxic to the human body [95]. Interestingly, Fe(III) and Cu(II) complexes with chlorogenic acid exhibit no cytotoxic activities on HaCaT cell lines at a 0.15-111 nM concentration [43]. In contrast, Mg(II) and Mn(II)/Na(I)––isoferulic acid complexes show significant cytotoxicity at concentrations of 16 μM and 45 μM, respectively, compared to free isoferulic acid, which only exhibits a 13% decrease in cell viability at a much higher concentration of 1582 μM [54]. This unexpected result points to the importance of differing mechanisms of action, stability, and cellular uptake in influencing the cytotoxic profiles of metal–phenolic complexes.

### 5.4. Genotoxic Activity

Genotoxicity of the complexes is a critical factor in assessing their potential application due to the risk of DNA damage, which can disrupt cellular metabolism and lead to cell death [96]. Matejczyk et al. evaluated the genotoxic activity of metal (Li(I), Na(I), and K(I)) complexes with caffeic and rosmarinic acid against bacteria [97]. The study found that rosmarinic acid, after coordination with the above-mentioned metals, induced a bacterial SOS response to DNA damage, as evidenced by the overexpression of a recA promoter–GFP construct. Similarly, Halevas et al. [98] observed that the frequency of sister chromatid exchange in Cu–chrysin complexes was enhanced compared to the control, demonstrating significant genotoxic activity against normal human lymphocytes. Metal–phenolic networks (MPNs) have been utilized for various cancer therapies such as photothermal therapy (PTT), chemodynamic therapy (CDT), and photodynamic therapy (PDT). They can accumulate in tumour sites and enhance therapeutic efficacy by generating reactive oxygen species (ROS) and disrupting cellular functions [99]. It should also be noted that chelation with phenolic compounds is commonly used in chelation therapy to counteract metal toxicity, owing to the strong affinity of phenolic compounds for metal ions. Phenolic compounds like tannic acid can mitigate metal toxicity through chelation and antioxidant mechanisms [100].

## 6. Conclusions and Future Perspectives

**Figure 3:**
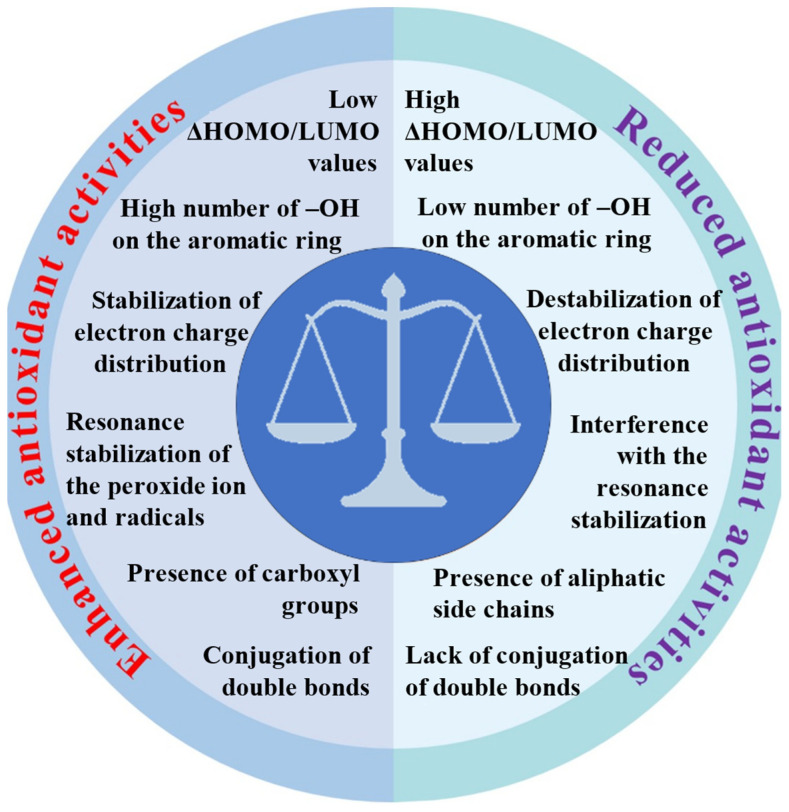
Relationship between molecular structure and antioxidant activities of metal–phenolic compounds.

We discussed and summarized the latest data from the world literature on the action of antioxidants. The review also summarizes our many years of experimental experience and the results of theoretical calculations. Our research employed a range of complementary methods, including spectroscopic techniques (FT-IR, FT-Raman, NMR, UV/Vis), X-ray diffraction, thermal analysis, quantum calculations, aromaticity index and descriptor analyses, and biological assays to assess antioxidant activity (DPPH, ABTS, FRAP, cytotoxicity, and genotoxicity tests). Our research confirmed global findings that the number and position of hydroxyl groups on aromatic rings are key factors in antioxidant effectiveness. This is largely because proton dissociation from the hydroxyl group plays a crucial role, especially in aqueous solutions. We have shown that the efficacy of antioxidants is also significantly influenced by other factors, such as the delocalization of the electron charge in aromatic rings and entire ligand molecules and the length of conjugated double bonds. Figure 3 summarizes the relationship between the molecular structure and antioxidant activities of metal–phenolic compounds.

The complexation of antioxidants with metals can have dramatically different effects on their effectiveness, with the outcomes varying widely, even having opposite effects depending on the metal involved. There are groups of metals that significantly increase antioxidant properties. Our long-term research shows that the potential of metals has the most significant impact on the antioxidant properties of ligands. Metals with high ionic potential measured by the charge-to-radius ratio [Fe(III), Cr(III), Ln(III), Y(III), Al(III), Mg(II), Cu(II), Zn(II)], stabilizing the distribution of the electron charge in complexes, increase the antioxidant properties of ligands. Metals with low ionic potential such as Ag(I), Hg(I), Hg(II), and Pb(II) disturb the electronic charge distribution and reduce antioxidant properties. However, it is essential to note that we did not always observe straightforward correlations between the ionic potential of metals and the increase in the antioxidant capacity of the ligands. The final effect, which is the increase in the effectiveness of antioxidants, also depends on other additional factors, as summarized below. The change in the molecular and electronic structure of ligands due to metal complexation significantly affects the antioxidant properties of ligands.

The study of metal–phenolic compound complexes (MPCCs) offers deep and profound insights into the interactions between metal ions and phenolic compounds.

We analyzed the changes in the molecular structure and electronic charge distribution after the coordination of ligands by metals based, among others, on the following criteria:

(1) Shifts in bathochromic or hypochromic bands π-> π* in UV/Vis absorption spectra.
(2) The decay or intensity of aromatic system bands (FT-IR, FT-Raman).
(3) Changes in the chemical shift δ of hydrogen and carbon atoms (1H and 13C NMR).
(4) Analyses of the crystallographic structure (X-ray).
(5) Composition, stability, and thermal durability (thermogravimetric analysis).
(6) Analyses of descriptors describing, among others, the distribution of electronic charge, bond lengths, and angles between bonds.
(7) Analyses of the size of differences between HOMO and LUMO energy levels (theoretical calculations).
(8) Analyses of aromaticity indices.

We examined the antioxidant activity using the following methods:

(1) Biochemical tests of antioxidant activity: DPPH, ABTS, and FRAP.
(2) Biological methods—MTT cytotoxicity, genotoxicity, and antimicrobial tests.

Our experiments were conducted in the so-called “logical” series of metals (e.g., Li(I)->Na(I)->K(I)->Rb(I)->Cs(I)), where the oxidation state was constant while the atomic radius varied, or in series (e.g., Li(I)->Ca(II)->La(III)->Th(IV)) where the oxidation state varied, but the atomic radius remained very similar. We also conducted studies in a logical series of ligands where each subsequent ligand differed from the previous one by the same functional group (e.g., hydroxyl group (OH)).

Our research confirmed findings from the global literature that a key factor determining the effectiveness of antioxidants is the number and position of hydroxyl groups attached to aromatic rings. However, we drew attention to the fact that the effectiveness of antioxidants is also significantly influenced by other factors, such as the delocalization of the electronic charge in aromatic rings and whole ligand molecules and the length of conjugated double bonds.

The complexation of ligands with metals has a diametrically different effect on the effectiveness of antioxidants.

Our research indicates that complexation with metals does not uniformly alter the antioxidant properties of ligands. This nuanced effect is influenced by metal parameters such as the oxidation state, ionic radius, and electronegativity, providing valuable insights into optimizing antioxidant performance.

Our long-term research shows that the most critical parameter influencing the electronic system’s degree of disturbance or stabilization is the ionic potential measured by the charge-to-radius ratio. At the same time, the ionic potential of metals has the most significant influence on the antioxidant properties of ligands. Metals with high ionic potential (such as Fe(III), Cr(III), Ln(III), Y(III), Al(III), Mg(II), Cu(II), and Zn(II)) and delocalized orbitals, stabilizing the distribution of the electronic charge, increase the antioxidant properties of ligands. Metals with low ionic potential, such as Ag(I), Hg(I), Hg(II), and Pb(II), disturb the distribution of the electronic charge and reduce the antioxidant properties of the ligand.

It should be emphasized that we only sometimes found simple correlations between the ionic potential of metals and the increase in the antioxidant properties of ligands. In some cases, metals with high ionic potential did not increase the antioxidant properties of ligands when complexing them. If such metals, when forming bonds with ligands, coordinated through OH groups and not through the carboxyl group, they reduced the antioxidant properties of these ligands. This phenomenon was observed, for example, in complexes with gallic acid.

**Figure 4:**
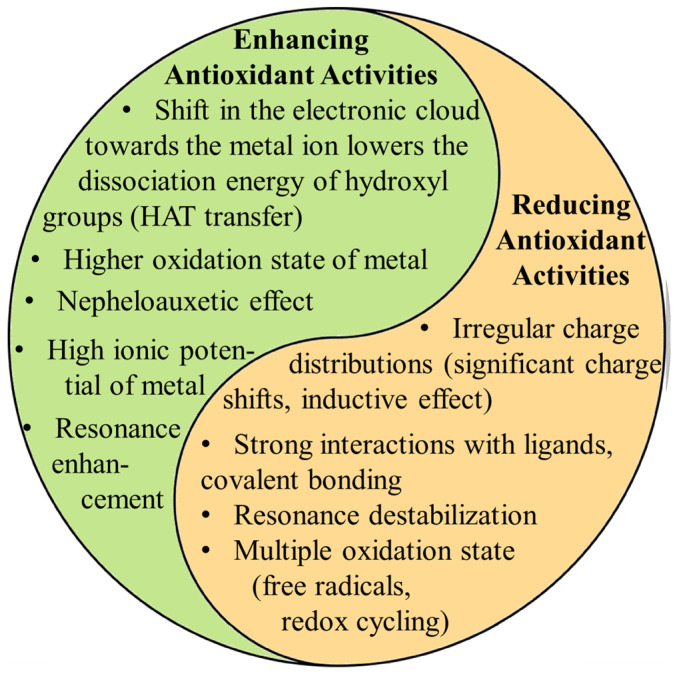
Highlights the dual role metal ions can play in influencing polyphenol antioxidant activity. Metals can enhance antioxidant activity by shifting electron density, lowering the energy required for hydrogen atom transfer (HAT), and promoting resonance stabilization. These factors facilitate the polyphenol’s ability to neutralize free radicals. On the other hand (right column), metals can reduce antioxidant activity through irregular charge distributions, covalent solid bonding, and resonance destabilization. The ultimate effect depends on the combination of the metal’s properties (oxidation state, charge distribution, redox potential, ionic radius, coordination number, electronegativity, and electron configuration) and its interaction with the ligand electronic structure.

The coordination of metal ions alters the distribution of electron charges on the aromatic ring, enhancing the dissociation of hydrogen ions from aromatic rings. However, their toxicity upon human digestion and the form of metal ions during this process are to be carefully investigated. Questions remain about potential adverse effects on organs if metal ions dissociate from the complexes. In vitro, antioxidant assays suggest that MPCCs are highly effective against free radicals since they are directly added to the solution. However, these assays may need to accurately reflect real-life effectiveness, as digestion and absorption in the human body will certainly limit their availability and chemical form, potentially reducing their biological effectiveness. Future research should focus on more extensive animal and clinical trials to verify the safety and efficacy of MPCCs, potentially accelerating their application in functional foods and pharmaceuticals. The low difference between the HOMO and LUMO indicates that MPCCs are likely to possess higher biological activity and reactivity than free ligands (Figure 3). Metal ion coordination has displayed promising results in enhancing the biological activities of phenolic compounds. However, many challenges remain that need to be addressed. Recently, in vitro studies suggested that MPCCs are non-toxic to human cells. Figure 4 summarizes metal ions’ role in influencing polyphenol antioxidant activity.

In summary, we conclude that the effectiveness of antioxidants depends not only on the number and position of hydroxyl groups but also on other factors, such as the degree of delocalization of bonds in complexes. Additionally, numerous instances have been documented where the same compound exhibits different effects, acting as an antioxidant at higher concentrations and as a pro-oxidant at lower concentrations.

## Data Availability

The original contributions presented in the study are included in the article/Supplementary Materials; further inquiries can be directed to the corresponding author.

## Supplementary Materials

The following supporting information can be downloaded at https://www.mdpi.com/article/10.3390/ijms252111775/s1. References [101,102,103,104,105,106,107,108,109] are cited in the Supplementary Materials.

## Abbreviations

ABTS	2,2′-Azino-bis(3-ethylbenzothiazoline-6-sulfonic acid) (radical scavenging assay)
APT	Atomic Polar Tensor
CDT	Chemodynamic Therapy
DPPH	2,2-Diphenyl-1-picrylhydrazyl (radical scavenging assay)
DSC	Differential Scanning Calorimetry
FRAP	Ferric Reducing Antioxidant Power
FT-IR	Fourier Transform Infrared Spectroscopy
FT-Raman	Fourier Transform Raman Spectroscopy
HOMO	Highest Occupied Molecular Orbital
LUMO	Lowest Unoccupied Molecular Orbital
MDPI	Multidisciplinary Digital Publishing Institute
MPCCs	Metal–Phenolic Compound Complexes
NBO	Natural Bond Orbital
NMR	Nuclear Magnetic Resonance
PPs	Polyphenolic Compounds
PDT	Photodynamic Therapy
PTT	Photothermal Therapy
SEM	Scanning Electron Microscopy
TEM	Transmission Electron Microscopy
TG	Thermogravimetric
UV/Vis	Ultraviolet–Visible Spectroscopy
XRD	X-Ray Diffraction
