# Supplementary material for: Exploring the Correlation Between the Molecular Structure and Biological Activities of Metal–Phenolic Compound Complexes: Research and Description of the Role of Metal Ions in Improving the Antioxidant Activities of Phenolic Compounds
Source: Int J Mol Sci. 2024 Nov 1;25(21):11775. doi: 10.3390/ijms252111775 (PMC11546605; doi:10.3390/ijms252111775)
Supplement: Supplementary file 1 [file ijms-25-11775-s001.zip › ijms-3256045-supplementary.pdf]

Exploring the correlation between molecular structure and biological activities of metal-phenolic compound complexes: Research and description of the role of metal ions in improving antioxidant activities of phenolic compounds

Zhe Chen[1], Renata Świsłocka[1,2], Renata Choińska[1], Krystian Marszałek[1], Aleksandra Dąbrowska[1], Włodzimierz Lewandowski[2], Hanna Lewandowska[3,4*]

[1] Prof. Wacław Dąbrowski Institute of Agricultural and Food Biotechnology – State Research Institute, Warsaw, Poland.

[2] Department of Chemistry Biology and Biotechnology, Bialystok University of Technology, Wiejska 45E, 15-351 Bialystok, Poland.

[3] School of Health & Medical Sciences, University of Economics and Human Sciences in Warsaw, Okopowa 59, 01-043, Warsaw, Poland.

[4] Centre for Radiation Research and Technology Institute of Nuclear Chemistry and Technology 16 Dorodna St., 03-195, Warsaw, Poland

*Authors to whom correspondence should be addressed

Table of contents.

Table number	Table title
Supplementary Table 1	Synthesis parameters of MPCCs
Supplementary Table 2	FITR spectra of phenolic compound-metal complexes.
Supplementary Table 3.	Changes in antioxidant and antimicrobial activities of the complexes

Supplementary Table 1. Synthesis parameters of MPCCs.

Name of phenolic compounds	Metal salt	Metal-phenolic compounds complexes	Synthesis parameters	Reference
2,4-Dihydroxybenzoic acid	TiF_4	2,4DHBA-Ti	Mixed in distilled water containing sodium acetate, 60 ℃, 4h, the molar ratio of 2,4DHBA to TiF_4 is 2:1	[1]
Gentisic acid (2,5-Dihydroxybenzoic acid)	LiOH	2,5DHBA-Li	Mixed in distilled water containing $CaCl_2$, a few days, the molar ratio of 2,5DHBA: Li is 1:1	[2]
2,6-Dihydroxybenzoic acid	TiF_4	2,6DHBA-Ti	Mixed in distilled water, 2h, the ratio of 2,6DHBA to TiF_4 is 2:1	[1]
2,6-dihydroxybenzoic acid	$NiCl_2$, $Co(NO_3)_2$	2,6DHBA-Ni, 2,6DHBA-Co	Mixed in distilled water containing a few drops of 2 M ammonia solution, pH 4, 60 ℃, 4h, the concentration ratio of 2,6DHBA:Ni or Co is 1:1	[3]
Protocatechuic acid (3,4-Dihydroxybenzoic acid)	Po	PCA-Po	Mixed in water, DMSO, ethanol, acetone, THF, diethyl-ether, gas	[4]
3,5-Dihydroxybenzoic acid	TiF_4	3,5DHBA-Ti	Mixed in distilled water, 2h, the ratio of 3,5DHBA to TiF_4 is 2:1	[1]

Gallic acid	$Zn(CH_3COO)_2$	GA-Zn	Mixed in methanol, the molar ratio of GA: $Zn(CH_3COO)_2$ is 2:1	[5]
Gallic acid	$CaCl_2$, $CuCl_2$, $CrCl_3$, $ZnCl_2$	GA-Ca, GA-Cu, GA-Cr, GA-Zn	Mixed in distilled water, 3h, the molar ratio of metal ions : GA is 1:1	[6]
Gallic acid and quercetin	$ZnSO_4$, $MnCl_2$	GA/QC-Zn, GA/QC-Mn	Mixed in methanol, 3h, room temperature, the mass ratio of GA:QC:metal ion is 1:1:2,	[5]
Caffeic acid	$EuCl_3$ $GdCl_3$, $DyCl_3$	CFA-Eu, CFA-Gd, CFA-Dy	Mixed in water, 50h, the stoichiometric ratio of NaCFA: metal ions is 3:1	[7,8]
Caffeic acid	LiOH, NaOH, KOH	CFA-Li, CFA-Na, CFA-K	Mixed in water, 70 °C, 1h, the stoichiometric ratio of NaCFA: metal ions is 1:1	[9]
ϱ-coumaric acid	$EuCl_3$, $GdCl_3$, $DyCl_3$	ϱ-CA-Eu, ϱ-CA-Gd, ϱ-CA-Dy	Mixed in water, 1h, the stoichiometric ratio of Naϱ-CA: metal ion is 3:1	[8]
Homovanillic acid	LiOH, NaOH, KOH, RbOH, CsOH	HVA-Li, HVA-Na, HVA-K, HVA-Rb, HVA-Cs	Mixed in water, condensed at 70 °C, the molar ratio of HVA: metal ion is 1:1	[10,11]
Isoferulic acid	$MgCl_2$	IFA-Mg	Mixed in water, 1h, the molar ratio of NaIFA: $MgCl_2$ is 1:1	[12]
o-Coumaric acid	LiOH, NaOH, KOH, RbOH, CsOH	o-CA-Li, o-CA-Na, o-CA-K, o-CA-Rb, o-CA-Cs	Mixed in water, condensed at 70 °C, the molar ratio of o-CA:metal ion is 1:1	[13]
Ferulic acid	$CaCl_2$, $CuCl_2$, $CdCl_2$, $ZnCl_2$, $MnCl_2$	FA-Ca, FA-Cu, FA-Cd, FA-Zn, FA-Mn	Mixed in water, 1h, the molar ratio of NaFA: metal ion is 1:2	[14]
Mandelic acid	LiOH, NaOH, KOH, RbOH, CsOH	MA-Li, MA-Na, MA-K, MA-Rb, MA-Cs	Mixed in water, 50 °C, 1h, the molar ratio of MA:metal ion is 1:1	[15]
Rosmarinic acid	LiOH, NaOH, KOH	RA-Li, RA-Na, RA-K	Mixed in water, 70 °C, 1h, the stoichiometric ratio of NaRA: metal ions is 1:1	[9,16]

Cichoric acid	$CuCl_2$, $CoCl_2$, $ZnCl_2$, $NiCl_2$	CA-Cu, CA-Co, CA-Zn, CA-Ni	Mixed in water, 25 °C, 2h, the stoichiometric ratio of NaCA: metal ions is 1:2	[17]
Chlorogenic acid	$FeCl_3$, $CuCl_2$	CQA-Fe, CQA-Cu	Mixed in water, the malor ratio of NaCQA: $FeCl_3$ and NaCQA: $CuCl_2$ is 1:2 and 1:3, respectively	[18]
Rutin	LiOH, NaOH, KOH, RbOH, CsOH	RT-Li, RT-Na, RT-K, RT-Rb, RT-Cs	Mixed in methanol, the malor ratio of RT: metal ions is 1:1	[19]
Chrysin	$CuCl_2$	CY-Cu	Mixed in methanol aqueous solution, 4h, the malor ratio of CY: $CuCl_2$ is 1:1	[20]
Quercetin	$CoCl_2$	QUE-Co	Mixed in methanol aqueous solution, pH 7.4, the malor ratio of CY: $CoCl_2$ is 1:2	[21]
Quercetin	$ZnCl_2$, $MnCl_2$, NaCl	QUE-Co, QUE-Co, QUE-Co	Mixed in methanol aqueous solution, Zn and Mn: pH 10, 1.5h, the malor ratio of QUE: $ZnCl_2$ or $MnCl_2$ is 1:2; Na: 1.5h, the malor ratio of QUE: NaCl is 1:1	[22]
Apigenin (API), chrysin (CHR), kaempferol (KAE) and quercetin (QUE)	$(CH_3COO)_2Pb$	API-Pb, CHR-Pb, KAE-Pb, QUE-Pb	Mixed in ethanol aqueous solution, 80 °C, 4h, the malor ratio of ligand: $(CH_3COO)_2Pb$ is 1:1	[23]
Hesperitin	$CuCl_2$	HSP-Cu	Mixed in ethanol aqueous solution, 12h	[24]

Supplementary Table 2. FITR spectra of phenolic compound-metal complexes.

	Phenolic compound	Metal	Findings	Reference
Phenolic acid	2,4-dihydroxybenzoic acid	Ti	ν(C=O) and ν(OH) shifted to lower frequencies	[1]
	2,5-dihydroxybenzoic acid (gentistic)	Ca	ν(OH), ν(C-C), ν(C-O), β(C-H), and α(C-C-C) shifted to higher or lower wavenumber, appearance of the asymmetric and symmetric vibrations of the carboxylate anion. disappearance of ν(C=O) from carboxylic group.	[2]
	2, 6- dihydroxybenzoic acid	Ti	ν(C=O) and ν(OH) shifted to lower frequencies	[1]
	3,5-dihydroxybenzoic acid	Ti	ν(C=O) and ν(OH) shifted to lower frequencies	[1]
	3,4,5-dihydroxybenzoic acid (Gallic acid)	Ca<br>Cu<br>Zn<br>Cr	ν(C=O) and ν(OH) shifted to lower frequencies, appearance of carboxylate anion ν_{as}(COO-) and ν_{s}(COO-),	[5,6]
Coumarin derivatives	o-coumaric acid	Li, Na and K	disappearance of stretching vibrations ν(OH), ν(C=O), β (OH), ν(C=O) and deformation vibrations β(O–H); appearance of bands of the asymmetric and symmetric vibrations of the carboxylate anion and disappearance or changes in positions and intensities of some aromatic bands.	[13]
	p-coumaric acids	Eu, Gd and Dy	disappearance of the bands assigned to the vibrations of the carbonyl group C=O and the hydroxyl group –OH.	[8]
	Caffeic acid	Eu	stretching vibrations of the aromatic ring hydroxyl groups reduced. bending vibration of the hydroxyl groups disappeared or reduced	[7]
	Caffeic acid	Eu, Gd and Dy	stretching vibrations of the C=O carbonyl group and the hydroxyl group -OH were not observed. strong bands derived from the stretches of the carboxylate anion appeared.	[8]

	Rosmarinic acid	Li, Na, K, Rb and Cs	appearance of the asymmetric and symmetric vibrations of the carboxylate anion ν_{as}(COO-), ν_{s}(COO-), deformation of β(OH) and γ(C-O) of the carbonyl group, disappearance of ν(OH) and changes or disappearance in the intensities and positions of some aromatic bands.	[16]
	Cichoric acid	Cu, Zn, Ni and Co	disappearance of the carboxyl group of the tartaric acid moiety, appearance of the stretching vibrations of carboxylate anion ν_{sym}(COO-), ν_{as}(COO-), β_{as}(COO-) and β_{s}(COO-). changes in the intensity and location of the caffeic acid moiety, ν(C–C), β(C-H), γ(C-H), deformation of in-plane and out-of-plane vibrations of the aromatic ring, β (OH) shifted toward lower wavenumbers, γ(OH) shifted toward higher wavenumber or disappeared	[17]
	Chlorogenic acid	Cu and Fe	appearance of carboxylate aion ν_{as}(COO-) and ν_{s}(COO-), disappearance of ν(C=O), catechol group shifted to lower wavenumber	[18]
	Ferulic Acid	Ca, Zn, Cu, Cd and Mn	disappearance of ν(C=O), $\gamma(OH)_{COOH}$, and γ(C=O) from carbonyl group, $\nu(OH)_{ar}$ and $\nu(C{=}C)_{-C=C-}$ shifted toward lower and higher wavenumber, respectively.	[14]
	Isoferulic Acid	Mg, and Mn/Na	disappearance of stretching vibrations ν(O–H) and ν(C=O), different locations of bands assigned to the vibrations of the ring -OH group, aromatic ring shift towards higher wavenumbers.	[12]
	homovanillic acid	Na	Disappearance of stretching vibrations of carboxyl group ν(OH) and ν(C=O) and deformations of β(C=O) and γ(C=O), appearance of carboxylate anion ν_{as}(COO-) and ν_{s}(COO-), deformation of β(COO-) and γ(COO-), and some changes of intensities and wavenumbers of the bands of aromatic system and methoxy, hydroxyl and CH_2 groups.	[11]
	homovanillic acid	Li, Na, K, Rb and Cs	disappearance of ν(OH) and deformation of β(OH), ν(C-O) and γ(C-O) of the carbonyl group are observed. appearance of bands of the asymmetric and symmetric vibrations of the carboxylate anion ν_{as}(COO-), ν_{s}(COO-) as well as β_{as}(COO-), β_{s}(COO-) and changes or disappearance in the intensities and positions of some aromatic bands	[10]
	Mandelic acid	Li, Na, K, Rb and Cs	appearance of the asymmetric and symmetric vibrations of the carboxylate anion ν_{as}(COO-) and ν_{s}(COO-). deformation of β_{s}(COO-), β_{as}(COO-) and γ_{s}(COO-), changes in the number, position, and intensity of the bands of the aromatic system.	[15]
Flavon	Chrysin	Zn	ν(C=O) stretching vibrations shifted to lower frequencies, ν(OH) stretching vibrations appear	[25]
	Chrysin	Ga	ν(C=O) stretching vibrations shifted to lower frequencies, appearance of ν(Ga–O) vibrations, and ν(C-O) and δ(OH) shifted to higher frequencies.	[26]

Chrysin	Pb	appearance of ν(Metal-O)	[23]
Apigenin	Pb	appearance of ν(Metal-O)	[23]
Kaempferol	Pb	ν(C-OH) shifted towards a lower wavenumber, ν(C-C) showed hypochromic effect, appearance of ν(Metal-O).	[23]
Quercetin	Pb	ν(C-OH) shifted towards a lower wavenumber, ν(C-C) showed hypochromic effect, appearance of ν(Metal-O).	[23]
Quercetin	Co	C=O was slightly shifted towards a lower wavenumber, ν(OH) and ν(C=O) vibrations moved towards lower wavenumbers, appearance of the stretching vibrations of the C-O catechol group.	[21]
Quercetin	Zn and Ni	stretching ν(OH), ν(C=O) and deforming β(C–OH) vibrations were moved toward lower wavenumbers, decrease in the wavenumbers of the aromatic ring vibrations, increase in the wavenumbers of ν(C–O–C) and occurrence of the ν(C–O) of the catechol group and metal-O vibration.	[22]
Quercetin	Cu	C=O and δ(OH) downshifted, ν(C–O–C) upshifted	[27]
Quercetin	Cu	C=O downshifted, C=C upshifted, appearance of metal-O vibration and deformation C-H, disappearance of C-O-C and $(OH)_{ar}$,	[28]
Quercetin	Zn	ν(C=O) stretching vibrations shifted to lower frequencies,	[25]
Hesperitin	Cu	appearance of ν(Metal-O), ν(C=O) stretching vibrations shifted to lower frequencies, $\nu(OH)_{ar}$ and ν(OH) shifted to higher wavenumber, C=C absorption peaks of the aromatic ring shifted to lower wavenumber,	[24]
Rutin	Li, Na, K, Rb and Cs	decrease in the wavenumbers ν(CH), ν(CC), and ν_{as}(C–C–O), the most visible movement ν(C = O), ν(C–OH), ν(C–O–C). shifted toward higher wavenumbers ν(CC), ν_{as}(O–C–C), ν(C–O–C).	[19]

Supplementary Table 3. Changes in antioxidant and antimicrobial activities of the complexes.

Phenolic compounds	Metal	Findings	Reference
Antioxidant activity			
2,6-dihydroxybenzoic	Ni, Co	Increasing the DPPH radical scavenging activity of the complexes	[3]
Gallic acid	Zn	Enhancing the ABST radical scavenging activity of the complexes	[5]
Gallic acid	Cr, Cu, Ca and Zn	Reducing the ABTS and ORAC radical scavenging activities of the complexes, expect for Zn-gallic acid complexes; Declining the DPPH radical scavenging activity of all complexes	[6]
Isoferulic acid	Mg, Mn/Na	Increasing the DPPH, ABTS, CUPRAC, and OH radical scavenging activities, lipid peroxidation inhibitory capacity and pro-oxidant of the Mg-isoferulic acid. Enhancing the biological activities of the Mn/Na-isoferulic acid complexes, except for ABTS radical scavenging activity and lipid peroxidation inhibitory capacity	[12]
Caffeic acid	Eu	Reducing the ABTS and DPPH radical scavenging activities, CUPRAC and FRAP of the complexes	[7]
Caffeic acid	Li, Na and K	Enhancing the DPPH radical scavenging activity of the complexes	[9]
Chlorogenic acid	Cu, Fe	Declining the DPPH, ABTS and OH radical scavenging activities of the complexes	[18]
Rosmarinic acid	Li, Na and K	Improving the DPPH radical scavenging activity and FRAP of the complexes	[16]
Rosmarinic acid	Li, Na and K	Improving the DPPH radical scavenging activity of the complexes	[16]
Hesperitin	Cu	Reducing the DPPH and O_2^- radical scavenging activity of the complexes	[24]
Quercetin	Cu	Enhancing the DPPH radical scavenging activity of the complexes	[27]
Quercetin	Zn	No Change in the DPPH radical scavenging activity of the complexes	[25]
Quercetin	Co	Increasing the DPPH radical scavenging activity of the complexes	[21]
Chrysin	Zn	Increasing the DPPH radical scavenging activity of the complexes	[25]
Rutin	Li, Na, K,	Declining the DPPH radical scavenging activity of Li-, Na- and K-rutin complexes, whereas improving the	[19]

	Rb, Cs	DPPH radical scavenging activity of Rb- and Cs-rutin complexes; Reducing the FRAP of all complexes, except for Rb-rutin complexes	
		Antimicrobial activity	
Mandelic acid	Li, Na and K	Activating the inhibition effect on the L. *monocytogenes*, S. *aureus*, E. *coli*, S. *typhimurium*, B. *subtilis*, L. *backii*, C. *albicans*, K. *mucilaginose* activities	[15]
o-coumaric acid	Li, Na, K, Rb and Cs	Increasing the degree of growth inhibition of all complexes for *Bacillus subtilis*, *Proteus vulgaris*, *Pseudomonas aeruginosa* and *Staphylococcus aureus*; Reducing the inhibition capacity of the complexes for *Escherichia coli*	[13]
Ferulic acid	Na, Cu, Zn and Cd	Enhancing the effect of growth inhibition for *Escherichia coli* and *Staphylococcus aureus* in the complexes	[14]
Caffeic acid	Eu	Increasing the antimicrobial activity of the complexes	[7]
Homovanillic acid	Li, Na, K, Rb and Cs	Reducing the growth inhibition of *Escherichia coli* (except for the Na-homovanillic acid complex and *Pseudomonas aeruginosa* in the complexes; Enhancing the degree of growth inhibition for *Baccillus subtilis, Candida albicans,* and *Proteus vulgaris* in the complexes	[10]
Chrysin	Cu	Increasing the antimicrobial activity of Cu-chrysin complexes	[20]
Rutin	Na	Declining the degree of growth inhibition for *Pseudomonas aeruginosa*, *Escherichia coli and Klebsiella oxytoca* in the complexes; Improving the effect of growth inhibition for *Bacillus subtilis* in the complexes	[19]
2,6-dihydroxybenzoic acid	Ni and Co	Enhancing the inhibition effect on the growth of *Escherichia coli* and *Staphylococcus aureus* in the complexes	[3]

References:

1. Aoues, I.; Chaieb, Z.; Zizi, Z.; Benhaliliba, M. Synthesis and Structural Study of Titanium (IV) Complexes Derivative from 2, 6-; 3, 5-and 2, 4-Dihydroxybenzoic Acid Molecular Modelling Approach. *Turkish Journal of Materials* **2021**, *6*.
2. Kalinowska, M.; Mazur, L.; Regulska, E.; Korczak, A.; Świderski, G.; Lewandowski, W. Calcium Complex of 2,5-Dihydroxybenzoic Acid (Gentisic Acid): Synthesis, Crystal Structure, and Spectroscopic Properties. *Journal of Coordination Chemistry* **2016**, *69*, 2415–2421, doi:10.1080/00958972.2016.1197389
3. Santoso, S.P.; Ismadji, S.; Angkawijaya, A.E.; Soetaredjo, F.E.; Go, A.W.; Ju, Y.H. Complexes of 2, 6-Dihydroxybenzoic Acid with Divalent Metal Ions: Synthesis, Crystal Structure, Spectral Studies, and Biological Activity Enhancement. *Journal of Molecular Liquids* **2016**, *221*, 617–623.
4. Krishna, P.N.; Muraleedharan, K. Metal Chelation Ability of Protocatechuic Acid Anion with 210Po84; a Theoretical Insight. *Computational and Theoretical Chemistry* **2023**, *1220*, 113996.
5. Motloung, D.M.; Mashele, S.S.; Matowane, G.R.; Swain, S.S.; Bonnet, S.L.; Noreljaleel, A.E.; Oyedemi, S.O.; Chukwuma, C.I. Synthesis, Characterization, Antidiabetic and Antioxidative Evaluation of a Novel Zn (II)-Gallic Acid Complex with Multi-Facet Activity. *Journal of Pharmacy and Pharmacology* **2020**, *72*, 1412–1426.
6. El-Megharbel, S.M.; Hamza, R.Z. Synthesis, Spectroscopic Characterizations, Conductometric Titration and Investigation of Potent Antioxidant Activities of Gallic Acid Complexes with Ca (II), Cu (II), Zn (III), Cr (III) and Se (IV) Metal Ions. *Journal of Molecular Liquids* **2022**, *358*, 119196.
7. Arciszewska, Ż.; Gama, S.; Kalinowska, M.; Świderski, G.; Świsłocka, R.; Gołębiewska, E.; Naumowicz, M.; Worobiczuk, M.; Cudowski, A.; Pietryczuk, A. Caffeic Acid/Eu (III) Complexes: Solution Equilibrium Studies, Structure Characterization and Biological Activity. *International Journal of Molecular Sciences* **2022**, *23*, 888.
8. Świderski, G.; Kalinowska, M.; Gołębiewska, E.; Świsłocka, R.; Lewandowski, W.; Kowalczyk, N.; Naumowicz, M.; Cudowski, A.; Pietryczuk, A.; Nalewajko-Sieliwoniuk, E. Structures, Antioxidant Properties, and Antimicrobial Properties of Eu (III), Gd (III), and Dy (III) Caffeinates and p-Coumarates. *Molecules* **2023**, *28*, 6506.
9. Matejczyk, M.; Świsłocka, R.; Golonko, A.; Lewandowski, W.; Hawrylik, E. Cytotoxic, Genotoxic and Antimicrobial Activity of Caffeic and Rosmarinic Acids and Their Lithium, Sodium and Potassium Salts as Potential Anticancer Compounds. *Advances in medical sciences* **2018**, *63*, 14–21.
10. Samsonowicz, M.; Kowczyk-Sadowy, M.; Piekut, J.; Regulska, E.; Lewandowski, W. Spectroscopic Characteristic (FT-IR, FT-Raman, UV, 1H and 13C NMR), Theoretical Calculations and Biological Activity of Alkali Metal Homovanillates. *Journal of Molecular Structure* **2016**, *1109*, 1–12.
11. Samsonowicz, M.; Kowczyk-Sadowy, M.; Regulska, E.; Lewandowski, W. Molecular Structure and Spectroscopic Analysis of Homovanillic Acid and Its Sodium Salt–NMR, FT-IR and DFT Studies. *Spectrochimica Acta Part A: Molecular and Biomolecular Spectroscopy* **2014**, *118*, 1068–1074.
12. Kalinowska, M.; Gołębiewska, E.; Mazur, L.; Lewandowska, H.; Pruszyński, M.; Świderski, G.; Wyrwas, M.; Pawluczuk, N.; Lewandowski, W. Crystal Structure, Spectroscopic Characterization, Antioxidant and Cytotoxic Activity of New Mg(II) and Mn(II)/Na(I) Complexes of Isoferulic Acid. *Materials* **2021**, *14*, 3236, doi:10.3390/ma14123236.
13. Kowczyk-Sadowy, M.; Świsłocka, R.; Lewandowska, H.; Piekut, J.; Lewandowski, W. Spectroscopic (FT-IR, FT-Raman, 1H-and 13C-NMR), Theoretical and Microbiological Study of Trans o-Coumaric Acid and Alkali Metal o-Coumarates. *Molecules* **2015**, *20*, 3146–3169.

14. Kalinowska, M.; Piekut, J.; Bruss, A.; Follet, C.; Sienkiewicz-Gromiuk, J.; Świsłocka, R.; Rzączyńska, Z.; Lewandowski, W. Spectroscopic (FT-IR, FT-Raman, 1H, 13C NMR, UV/VIS), Thermogravimetric and Antimicrobial Studies of Ca (II), Mn (II), Cu (II), Zn (II) and Cd (II) Complexes of Ferulic Acid. *Spectrochimica Acta Part A: Molecular and Biomolecular Spectroscopy* **2014**, *122*, 631–638.
15. Świsłocka, R.; Świderski, G.; Nasiłowska, J.; Sokołowska, B.; Wojtczak, A.; Lewandowski, W. Research on the Electron Structure and Antimicrobial Properties of Mandelic Acid and Its Alkali Metal Salts. *International Journal of Molecular Sciences* **2023**, *24*, 3078.
16. Świsłocka, R.; Regulska, E.; Karpińska, J.; Świderski, G.; Lewandowski, W. Molecular Structure and Antioxidant Properties of Alkali Metal Salts of Rosmarinic Acid. Experimental and DFT Studies. *Molecules* **2019**, *24*, 2645.
17. Świderski, G.; Jabłońska-Trypuć, A.; Kalinowska, M.; Świsłocka, R.; Karpowicz, D.; Magnuszewska, M.; Lewandowski, W. Spectroscopic, Theoretical and Antioxidant Study of 3d-Transition Metals (Co (II), Ni (II), Cu (II), Zn (II)) Complexes with Cichoric Acid. *Materials* **2020**, *13*, 3102.
18. Kalinowska, M.; Gryko, K.; Gołębiewska, E.; Świderski, G.; Lewandowska, H.; Pruszyński, M.; Zawadzka, M.; Kozłowski, M.; Sienkiewicz-Gromiuk, J.; Lewandowski, W. Fe (III) and Cu (II) Complexes of Chlorogenic Acid: Spectroscopic, Thermal, Anti-/Pro-Oxidant, and Cytotoxic Studies. *Materials* **2022**, *15*, 6832.
19. Samsonowicz, M.; Kamińska, I.; Kalinowska, M.; Lewandowski, W. Alkali Metal Salts of Rutin–Synthesis, Spectroscopic (FT-IR, FT-Raman, UV–VIS), Antioxidant and Antimicrobial Studies. *Spectrochimica Acta Part A: Molecular and Biomolecular Spectroscopy* **2015**, *151*, 926–938.
20. Zhang, S.; Sadhasivam, D.R.; Soundarajan, S.; Shanmugavel, P.; Raji, A.; Xu, M. In Vitro and in Vivo Investigation of Chrysin Chelated Copper Complex as Biocompatible Materials for Bone Tissue Engineering Applications. *3 Biotech* **2023**, *13*, 45.
21. Kalinowska, M.; Lewandowska, H.; Pruszyński, M.; Świderski, G.; Gołębiewska, E.; Gryko, K.; Braun, J.; Borkowska, M.; Konieczna, M.; Lewandowski, W. Co (II) Complex of Quercetin–Spectral, Anti-/pro-Oxidant and Cytotoxic Activity in HaCaT Cell Lines. *Applied Sciences* **2021**, *11*, 9244.
22. Kalinowska, M.; Świderski, G.; Matejczyk, M.; Lewandowski, W. Spectroscopic, Thermogravimetric and Biological Studies of Na (I), Ni (II) and Zn (II) Complexes of Quercetin. *Journal of Thermal Analysis and Calorimetry* **2016**, *126*, 141–148.
23. Wang, Q.; Zhao, H.; Zhu, M.; Gao, L.; Cheng, N.; Cao, W. Spectroscopy Characterization, Theoretical Study and Antioxidant Activities of the Flavonoids-Pb (II) Complexes. *Journal of Molecular Structure* **2020**, *1209*, 127919.
24. Liu, K.; Zeng, N.; Pan, J.; Gong, D.; Zhang, G. Synthesis, Characterization, Toxicity Evaluation and Inhibitory Effect of Hesperitin-Copper (II) Complex on Xanthine Oxidase. *Journal of Molecular Liquids* **2022**, *368*, 120812.
25. Halevas, E.; Mavroidi, B.; Pelecanou, M.; Hatzidimitriou, A.G. Structurally Characterized Zinc Complexes of Flavonoids Chrysin and Quercetin with Antioxidant Potential. *Inorganica Chimica Acta* **2021**, *523*, 120407.
26. Halevas, E.; Mavroidi, B.; Antonoglou, O.; Hatzidimitriou, A.; Sagnou, M.; Pantazaki, A.A.; Litsardakis, G.; Pelecanou, M. Structurally Characterized Gallium–Chrysin Complexes with Anticancer Potential. *Dalton Transactions* **2020**, *49*, 2734–2746.
27. Halevas, E.; Pekou, A.; Papi, R.; Mavroidi, B.; Hatzidimitriou, A.G.; Zahariou, G.; Litsardakis, G.; Sagnou, M.; Pelecanou, M.; Pantazaki, A.A. Synthesis, Physicochemical Characterization and Biological Properties of Two Novel Cu (II) Complexes Based on Natural Products Curcumin and Quercetin. *Journal of inorganic biochemistry* **2020**, *208*,

111083.
28. Vimalraj, S.; Rajalakshmi, S.; Preeth, D.R.; Kumar, S.V.; Deepak, T.; Gopinath, V.; Murugan, K.; Chatterjee, S. Mixed-Ligand Copper (II) Complex of Quercetin Regulate Osteogenesis and Angiogenesis. *Materials Science and Engineering: C* **2018**, *83*, 187–194.
